# Ultrasound-Guided Synovial Biopsy in Chronic Knee Monoarthritis: A Case Series Highlighting Its Diagnostic Value

**DOI:** 10.7759/cureus.107378

**Published:** 2026-04-20

**Authors:** Siham Rachidi, Saadia Ait Malek, Imad Ghozlani, Mariam Erraoui

**Affiliations:** 1 Rheumatology, Mohammed IV University Hospital Center, Agadir, MAR; 2 Rheumatology, Oued Eddahab Military Hospital, Agadir, MAR; 3 Rheumatology, Cartilage and Bone (CARBONE) Research Team, Research Laboratory of Innovation in Health Sciences (LARISS), Faculty of Medicine and Pharmacy of Agadir, Ibn Zohr University, Agadir, MAR

**Keywords:** chronic monoarthritis, osteoarticular tuberculosis, pigmented villonodular synovitis, synovial biopsy, ultrasound-guidance

## Abstract

Ultrasound-guided synovial biopsy represents a valuable diagnostic tool in the investigation of chronic knee monoarthritis, particularly in cases where conventional clinical, laboratory, and imaging findings remain inconclusive. We report a case series of three patients presenting with chronic knee monoarthritis evolving over several months who were managed in a tertiary rheumatology center. In all cases, ultrasound-guided synovial biopsy was performed after inconclusive routine investigations and enabled definitive etiological diagnosis. Histopathological examination revealed two cases of osteoarticular tuberculosis and one case of localized pigmented villonodular synovitis, thereby allowing timely and targeted therapeutic management. This case series highlights the diagnostic accuracy, safety, and reproducibility of this minimally invasive technique and emphasizes its potential role in reducing diagnostic delays and improving patient outcomes.

## Introduction

Chronic knee monoarthritis represents a significant diagnostic challenge in rheumatology practice when clinical evaluation, laboratory investigations, synovial fluid analysis, and imaging findings remain inconclusive. In such circumstances, synovial biopsy plays a pivotal role in such situations, particularly when neoplastic or infectious etiologies are suspected, including mycobacterial or other intracellular pathogens. By providing definitive histological and/or microbiological confirmation, synovial biopsy facilitates early diagnosis and guides appropriate therapeutic intervention.

In recent years, musculoskeletal ultrasound has emerged as an essential tool in the assessment of rheumatic diseases, enabling accurate evaluation of synovial hypertrophy, inflammatory activity, and associated structural joint abnormalities. When combined with real-time procedural guidance, ultrasound significantly improves the accuracy, diagnostic yield, and safety of synovial biopsy [1].

The present case series aims to illustrate the diagnostic value of ultrasound-guided synovial biopsy in patients with chronic knee monoarthritis and to highlight its impact on etiological diagnosis and subsequent therapeutic management.

## Case presentation

We report a case series of three patients managed in the Rheumatology Department, selected based on the presence of chronic knee monoarthritis evolving for approximately one year and refractory to initial medical management.

Case 1

A 31-year-old male patient with no significant family history was referred for evaluation of a refractory right knee monoarthritis. His past medical history was notable for a left elbow fracture treated by traditional non-medical methods in 2002 and a prior history of tobacco and alcohol use, both discontinued two years before presentation. The patient had been under rheumatological follow-up for seronegative destructive chronic inflammatory arthritis. Over the course of his disease, significant structural damage had accumulated, including bilateral hip involvement, right carpal fusion, and a fixed flexion deformity of the left elbow. Notably, sacroiliac joint involvement was absent on imaging. He had been receiving methotrexate at a dose of 15 mg per week and had achieved sustained clinical remission prior to the current presentation.

The patient presented with an acute inflammatory flare manifested by progressive right knee swelling. Physical examination demonstrated an antalgic gait and isolated right knee monoarthritis. Additional pain was elicited at the left ankle and left elbow. Articular range of motion was globally reduced in the right knee, right hip, right wrist, and left elbow. Axial assessment revealed significant spinal mobility impairment, with a Schober index of 3.5 cm and a finger-to-floor distance of 47 cm. The clinical picture evolved in the context of general health deterioration associated with constitutional night sweats, raising concern for a systemic or infectious etiology.

**Figure 1:**
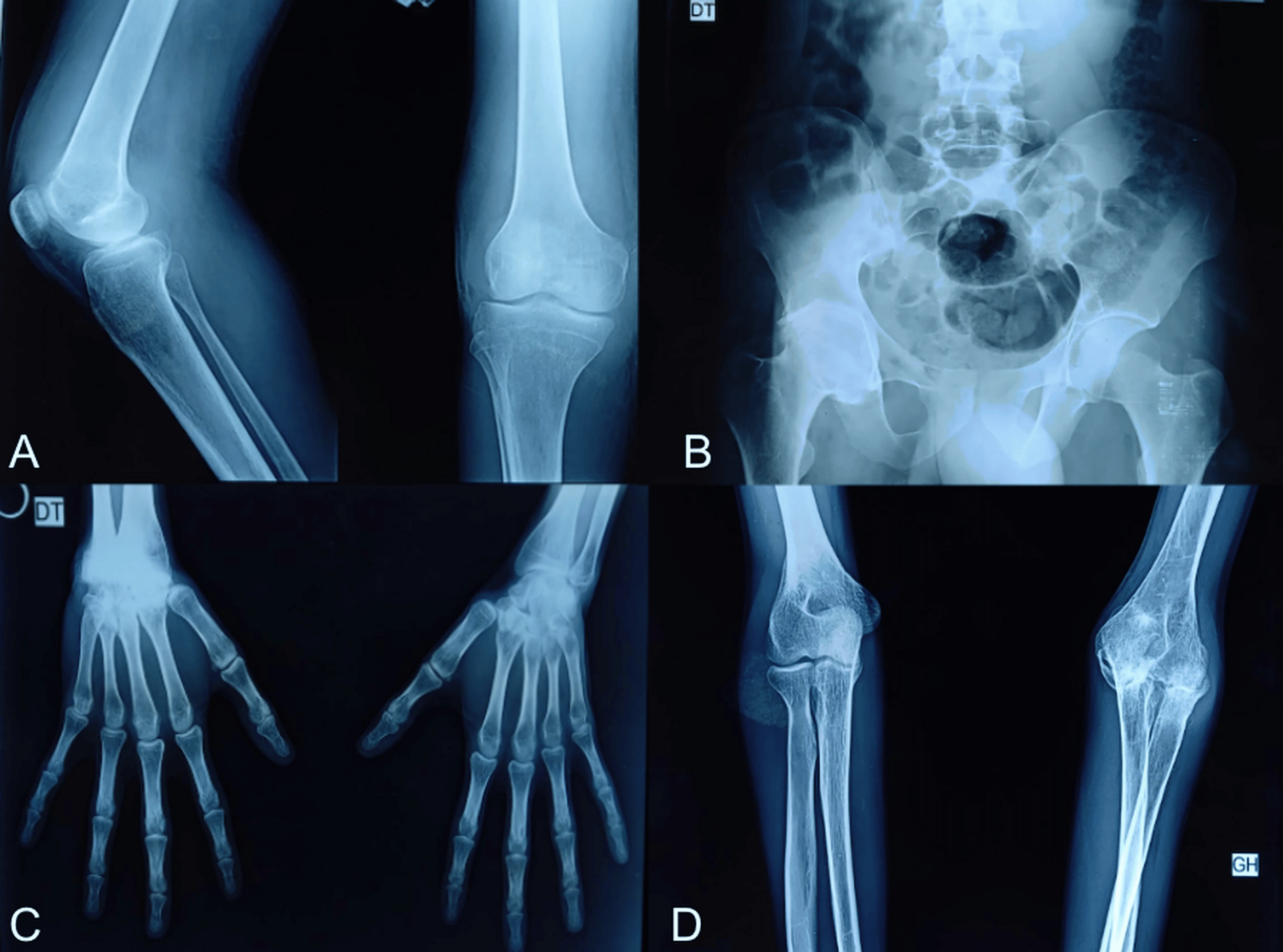
Conventional radiograph findings Demonstrating advanced structural damage of the right knee with joint space narrowing (A), bilateral hip involvement (B), right carpal fusion (C), and fixed flexion deformity of the left elbow (D).

Laboratory workup demonstrated a marked inflammatory response, with an erythrocyte sedimentation rate (ESR) of 50 mm/h (reference range: 0-20 mm/h) and a C-reactive protein (CRP) level of 65.7 mg/L (reference range: <5 mg/L). Synovial fluid analysis revealed an inflammatory effusion containing 12,000 cells/mm³ (reference range: inflammatory effusion 2,000-50,000 cells/mm³), with a neutrophil predominance. Musculoskeletal ultrasound of the right knee showed diffuse synovial hypertrophy with a mild degree of joint effusion. Conventional radiographs demonstrated advanced structural damage of the right knee, bilateral hip involvement with articular space narrowing, right carpal fusion, and fixed deformity of the left elbow (Figure 1).

**Figure 2:**
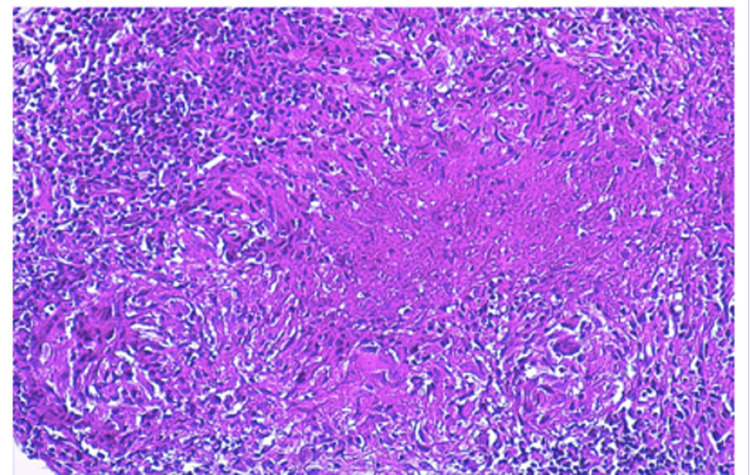
Hematoxylin-eosin stain of the synovial biopsy specimen Showing an epithelioid and giant-cell granuloma with central caseous necrosis, consistent with osteoarticular tuberculosis.

An initial intra-articular corticosteroid injection was administered, yielding transient clinical improvement followed by rapid symptomatic recurrence. In view of persistent refractory inflammatory monoarthritis in the context of ongoing methotrexate therapy, an ultrasound-guided synovial biopsy was undertaken. Histopathological examination of the synovial specimen demonstrated the presence of epithelioid and giant-cell granulomas with central caseous necrosis, a pathognomonic finding of mycobacterial infection, thereby confirming the diagnosis of osteoarticular tuberculosis (Figure 2).

Standard four-drug antituberculous therapy comprising isoniazid, rifampicin, pyrazinamide, and ethambutol was promptly initiated in accordance with national guidelines, with methotrexate temporarily withheld. Subsequent clinical and biological follow-up demonstrated favorable outcomes.

Case 2

An 11-year-old male with no prior significant medical history presented with a one-year history of chronic right knee monoarthritis refractory to symptomatic management. Of particular epidemiological relevance, his father had been treated for pulmonary tuberculosis over a period of two years, with reportedly poor medication adherence, thereby constituting a significant household exposure risk.

Physical examination revealed tenderness and restricted range of motion of the right knee, accompanied by moderate joint effusion. The remainder of the musculoskeletal examination was unremarkable, with no other joint involvement or extra-articular manifestations identified. The patient was afebrile, and his general condition was preserved.

Laboratory investigations disclosed a mild inflammatory response, with an ESR of 26 mm/h. Immunological screening for inflammatory arthropathy was negative. Tuberculin skin testing (TST) yielded a positive result with an induration of 12 mm, and the interferon-gamma release assay (IGRA) was likewise positive, together indicating sensitization to Mycobacterium tuberculosis. Plain radiography of the right knee revealed chronic monoarticular changes with involvement of the growth cartilage, consistent with the patient's age and disease duration.

**Figure 3:**
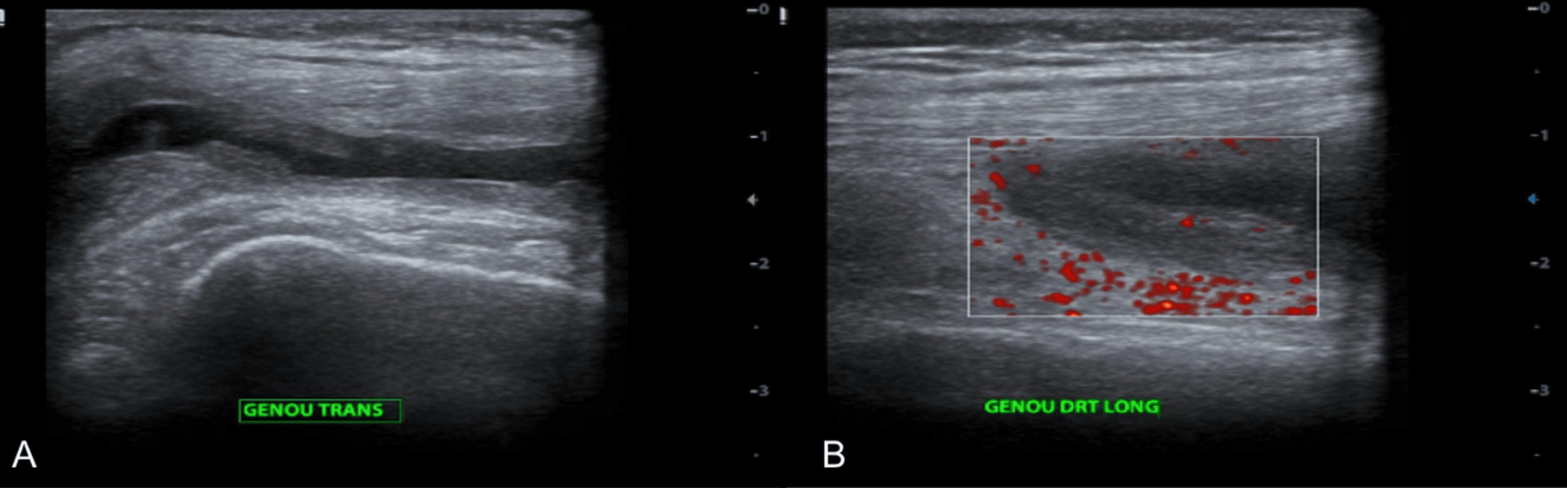
Musculoskeletal ultrasound of the right knee Demonstrating grade III chronic synovitis (A) with a negative Doppler signal (B), synovial hypertrophy with synovial fringes.

Synovial fluid analysis demonstrated a non-inflammatory appearance with negative cytobacteriological examination, sterile culture results, and no acid-fast bacilli identified on direct microscopy. Musculoskeletal ultrasound of the right knee showed grade III chronic synovitis without Doppler hypervascularity, associated with marked synovial hypertrophy, synovial fringes, and mild joint effusion (Figure 3).

Given the persistence of symptoms, the absence of a definitive etiological diagnosis on conventional investigations, and the highly suggestive epidemiological context of prolonged household exposure to active pulmonary tuberculosis, an ultrasound-guided synovial biopsy was performed. Histopathological examination demonstrated necrotizing tuberculoid granulomatous synovitis, establishing the diagnosis of osteoarticular tuberculosis. Antituberculous therapy was initiated, with gradual subsequent clinical and radiological improvement documented at follow-up.

Case 3

A 17-year-old female patient with a documented history of three previous self-limited episodes of left knee arthritis was admitted on suspicion of septic arthritis of the left knee. No relevant family history or prior chronic illness was identified.

Physical examination revealed a painful left knee with a fixed flexion deformity estimated at 20 degrees and moderate joint effusion, without florid inflammatory signs or overlying erythema. The patient was afebrile, and her general condition was preserved. No other articular or systemic manifestations were noted.

Laboratory investigations demonstrated hypochromic microcytic anemia consistent with iron deficiency, alongside leukocytosis with neutrophil predominance. A biological inflammatory syndrome was present, with an ESR of 41 mm/h (reference range: 0-20 mm/h) and a CRP level of 37.5 mg/L (reference range: <5 mg/L). Immunological testing for inflammatory rheumatic disease, including rheumatoid factor, antinuclear antibodies, and anti-cyclic citrullinated peptide antibodies, was entirely negative. The infectious workup, encompassing urine cytobacteriological examination, chest radiography, and blood cultures, was unremarkable. Plain radiography of the left knee demonstrated articular space sclerosis without erosive changes.

**Figure 4:**
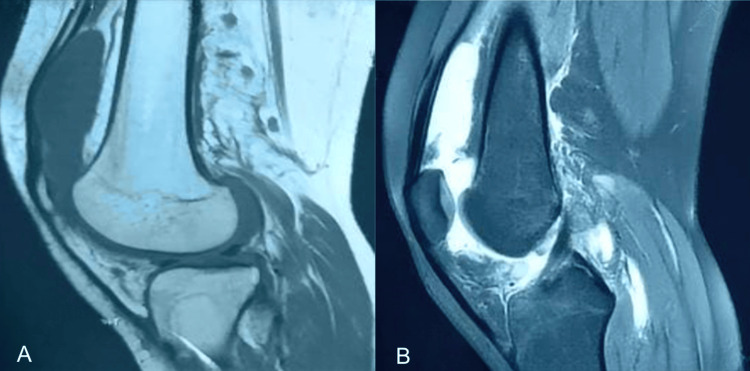
Magnetic resonance imaging of the left knee in sagittal planes Showing diffuse synovial thickening with low signal intensity on T1-weighted (A) and T2-weighted (B) sequences, associated with hemosiderin deposition, in keeping with pigmented villonodular synovitis.

Synovial fluid analysis revealed a purulent-appearing effusion with high cellularity and a neutrophil predominance of 90%. GeneXpert molecular testing performed on the synovial fluid was negative for Mycobacterium tuberculosis. Musculoskeletal ultrasound demonstrated chronic synovial hypertrophy with intra-articular septations and a positive Doppler signal. Magnetic resonance imaging of the left knee showed synovial thickening exhibiting low signal intensity on both T1- and T2-weighted sequences, with associated hemosiderin deposition, findings highly suggestive of pigmented villonodular synovitis (Figure 4).

**Figure 5:**
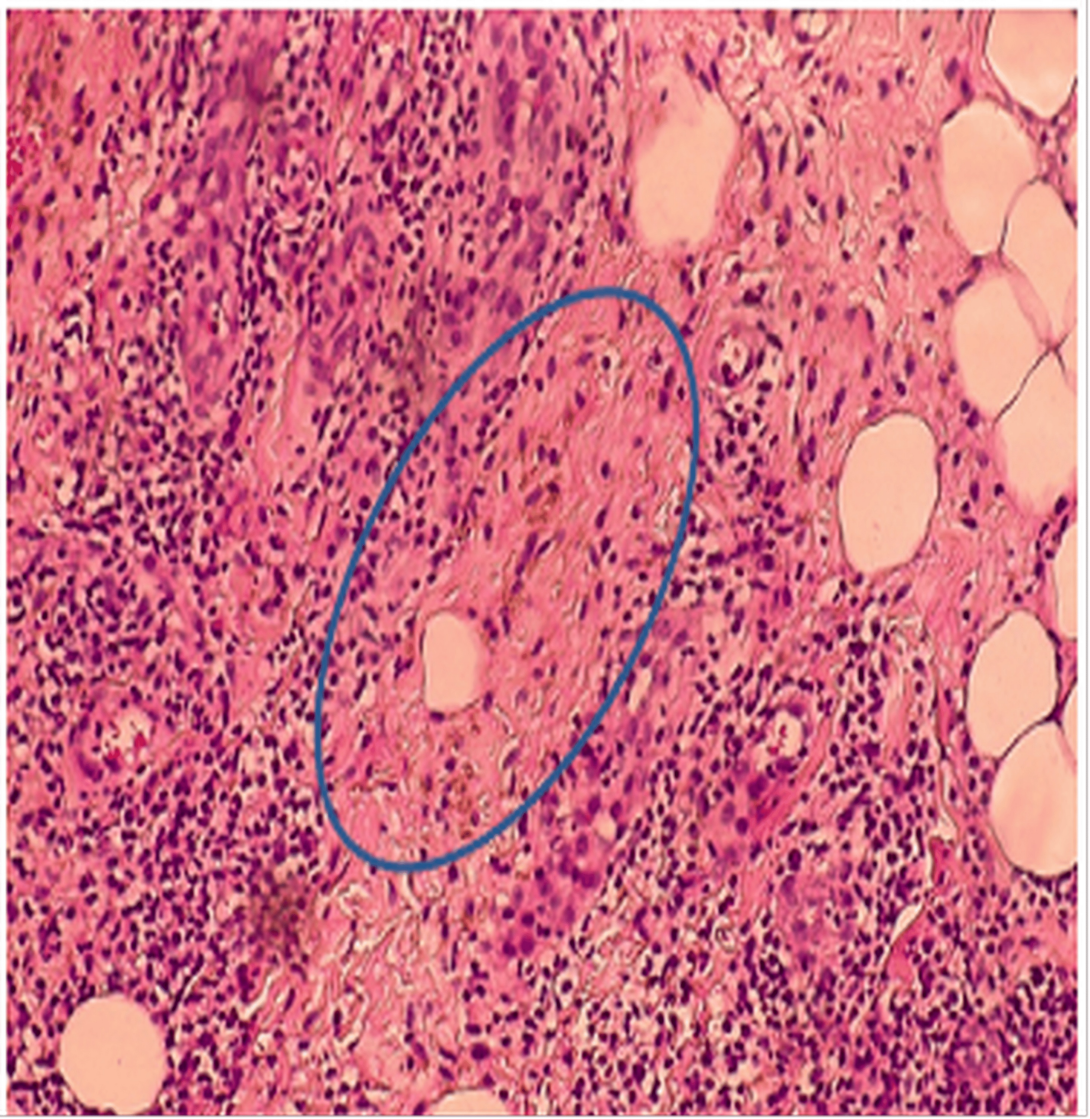
Histopathological examination Showing abundant hemosiderin deposits (circle).

Despite partial clinical improvement under empirical broad-spectrum antibiotic therapy, diagnostic uncertainty persisted; ultrasound-guided synovial biopsy was therefore performed. Histopathological examination definitively excluded tuberculous involvement and confirmed the diagnosis of focal pigmented villonodular synovitis, demonstrating characteristic villous synovial proliferation with multinucleated giant cells, hemosiderin-laden macrophages, and fibrosis (Figure 5). In view of the persistent symptomatology and the extent of synovial involvement, arthroscopic surgical synovectomy was proposed as the definitive therapeutic intervention.

## Discussion

Ultrasound-guided synovial biopsy has emerged as a reliable, minimally invasive, and reproducible diagnostic technique in the evaluation of chronic monoarthritis, particularly in clinical scenarios where physical examination, laboratory investigations, and conventional imaging fail to yield a definitive etiological diagnosis [1]. The present case series illustrates the broad diagnostic spectrum of this approach, demonstrating its decisive contribution in establishing a histologically confirmed diagnosis: osteoarticular tuberculosis in two patients and a benign tumoral lesion of the synovium in the third.

Compared with synovial fluid analysis alone, ultrasound-guided synovial biopsy affords the advantage of combined histopathological and microbiological evaluation, thereby substantially increasing diagnostic yield. This dual assessment enables accurate differentiation between infectious, inflammatory, and tumoral etiologies, minimizing the risk of diagnostic error and inappropriate therapeutic management. Its contribution is particularly valuable in atypical clinical presentations, including pediatric and adolescent cases, in which clinical and biological features are frequently nonspecific and may mimic a wide range of conditions [2].

From a technical standpoint, real-time ultrasound guidance enhances procedural accuracy by enabling direct visualization of synovial hypertrophy and vascular distribution, while simultaneously reducing the risk of procedural complications. The technique is well tolerated under local anesthesia, can be performed on an outpatient basis, and carries a low overall complication rate. Adherence to standardized procedural protocols, encompassing strict aseptic technique, targeted sampling of hypertrophic synovial tissue, and systematic collection of multiple biopsy specimens, further optimizes diagnostic performance [3].

In the context of suspected osteoarticular tuberculosis, synovial biopsy demonstrates markedly superior diagnostic sensitivity compared with synovial fluid analysis alone. Published data report sensitivities of approximately 85% and specificities approaching 95%, reflecting the technique's particular value in paucibacillary forms of the disease, where synovial fluid cultures are frequently negative and clinical suspicion may be the sole diagnostic driver [4-6].

Beyond infectious etiologies, synovial biopsy plays a pivotal role in the investigation of chronic inflammatory synovitis of undetermined origin. Specific histopathological patterns may orient the clinician toward defined inflammatory rheumatic conditions. In a landmark series, Kelly et al. [7] reported that synovial biopsy contributed to diagnostic reclassification in nearly 60% of cases of undifferentiated chronic synovitis, underscoring its utility as a second-line diagnostic tool in this setting.

Pigmented villonodular synovitis is a rare but potentially destructive proliferative disorder of the synovium that warrants early recognition. It may involve articular synovium or tendon sheaths and predominantly affects young adults between the third and fifth decades of life [8]. Its etiopathogenesis remains incompletely understood; two principal hypotheses have been proposed: a reactive inflammatory process secondary to recurrent microtrauma, and a neoplastic process associated with specific cytogenetic abnormalities, including translocation of the COL6A3-CSF1 gene [9]. Two distinct clinicopathological forms are recognized: a localized (nodular) variant, typically responsible for mechanical symptoms such as joint locking or a palpable mass, and a diffuse form characterized by chronic pain and recurrent, frequently hemorrhagic, joint effusions. Although magnetic resonance imaging provides highly characteristic signal findings, most notably low signal intensity on T1- and T2-weighted sequences attributable to hemosiderin deposition, histological confirmation remains mandatory prior to definitive surgical or pharmacological management [10]. Synovial biopsy is therefore indispensable not only to establish the diagnosis but also to guide therapeutic planning and to exclude alternative causes of chronic monoarthritis with overlapping clinical and imaging features [11].

Taken together, the findings of this case series reinforce the position of ultrasound-guided synovial biopsy as a cornerstone of the diagnostic algorithm for chronic monoarthritis. By improving etiological accuracy, shortening diagnostic delays, and preventing the institution of inappropriate therapeutic strategies, this technique should be considered early in the workup of any patient presenting with unexplained chronic joint disease that is unresponsive to empirical management [12].

## Conclusions

Ultrasound-guided synovial biopsy represents an essential diagnostic modality in the systematic evaluation of chronic knee monoarthritis. By yielding definitive histopathological and microbiological diagnoses, this technique enables reliable exclusion of infectious and neoplastic etiologies while providing the etiological basis required for targeted, evidence-based therapeutic management. Its early integration into the diagnostic algorithm significantly reduces diagnostic delays, limits unnecessary empirical treatments, and ultimately improves patient outcomes.
